# Adaptable robots, ethics, and trust: a qualitative and philosophical exploration of the individual experience of trustworthy AI

**DOI:** 10.1007/s00146-024-01938-8

**Published:** 2024-04-23

**Authors:** Stephanie Sheir, Arianna Manzini, Helen Smith, Jonathan Ives

**Affiliations:** Centre for Ethics in Medicine, University of Bristol, Bristol, UK

**Keywords:** Artificial intelligence, Trustworthy AI, Public perceptions of AI, AI ethics, Algorithmic accountability, AI governance

## Abstract

Much has been written about the need for trustworthy artificial intelligence (AI), but the underlying meaning of trust and trustworthiness can vary or be used in confusing ways. It is not always clear whether individuals are speaking of a technology’s trustworthiness, a developer’s trustworthiness, or simply of gaining the trust of users by any means. In sociotechnical circles, trustworthiness is often used as a proxy for ‘the good’, illustrating the moral heights to which technologies and developers ought to aspire, at times with a multitude of diverse requirements; or at other times, no specification at all. In philosophical circles, there is doubt that the concept of trust should be applied at all to technologies rather than their human creators. Nevertheless, people continue to intuitively reason about trust in technologies in their everyday language. This qualitative study employed an empirical ethics methodology to address how developers and users define and construct requirements for trust throughout development and use, through a series of interviews. We found that different accounts of trust (rational, affective, credentialist, norms based, relational) served as the basis for individual granting of trust in technologies and operators. Ultimately, the most significant requirement for user trust and assessment of trustworthiness was the accountability of AI developers for the outputs of AI systems, hinging on the identification of accountable moral agents and perceived value alignment between the user and developer’s interests.

## Introduction

### Background

Artificial intelligence (AI) is increasingly spoken of in the language of trust and trustworthiness, both in academic and policy vernacular (Brundage et al. 2020; Department for Science, Innovation and Technology 2023; Independent High-Level Expert Group on Artificial Intelligence 2019; Mökander and Floridi 2021). Corporations also state their commitment to trustworthy AI. While terms such as ‘safe’, ‘trustworthy’, and ‘responsible’ are used interchangeably, it is often unclear what they mean and what form of ‘good’ they describe.

The meaning of trust seems to vary widely with usage. It can be used, variously, to convey mutual feeling between people, for whom a breach of trust constitutes betrayal; a reliance on the continuing functioning and performance of someone or something, for which a breach of trust is not a betrayal but more a disappointment; or something in between (Ryan 2020). It is not always clear where placing trust in technology lies on this spectrum. One possible interpretation of trust in technology is *reliance*.

Relying on a technology involves placing confidence in it to carry out a particular action (Ryan 2020). Seeing a technology perform reliably over time can lead to a type of naïve trust. We characterize this trust as naïve because it is based solely on the inductive principle that future events will be like past events. Trust becomes less naïve when that empirically observed uniformity in performance over time is accompanied by some form of theoretical understanding, which gives us reason and confidence to believe a system will continue to act as it has done. However, it is not clear that reducing ‘trust’ to ‘reliance’ captures what is really intended when the language of trust in technology is used, or that it is reasonable to use them synonymously; and several epistemic confusions around the use of trust language require resolving.

First, there exists a suite of concerns relating to the relationship between trust, accountability, and moral agency, which can generally be termed anthropocentric objections (Freiman 2022)*.* These objections distinguish between trust and reliance (Baier 1986; Braun et al. 2021; Coeckelbergh 2012; Hawley 2014; Ryan 2020; Tallant 2019). While reliance simply involves observation and prediction, trust is said to inhabit the inherently human territory of shared agreements between trustor and trustee, the placing and acceptance of moral responsibility for actions in a particular domain, the acknowledgment of agency and (free) will of the trustee, and emotional qualities such as the goodwill, real or perceived, of the trustee to the trustor (Jones 1996).

A second concern stems from the trust–trustworthiness gap. ‘Trust’ and ‘trustworthy’ are often used interchangeably, particularly by political and corporate actors, with varying meanings and implications for action. For instance, the drive for trustworthy AI can mean a technology which possesses attributes that are deserving of trust, which implies the need for safe, well-functioning systems; or it can mean a sociotechnical system possessing these attributes, implying also the existence of accountable, beneficent human creators and operators (Smith et al. 2023a, b). It can, however, mean something else entirely, for example the elicitation of trust as a phenomenon in consumer psychology, such as gaining the confidence of users through reputational campaigns and demonstrations of corporate goodwill (Lewis and Marsh 2022; Reinhardt 2023).

Furthermore, trustworthiness is also, at times, used as a proxy for ‘the good’—which highlights its moral connotations. Denoting a person as ‘trustworthy’ is not only a description of a trait, but also an evaluation of their character. A trustworthy person is understood to be a good person who will choose to do the right thing; otherwise, they are merely reliable or dependable.

### Autonomous systems with evolving functionality (ASEFs)

Trust is particularly difficult to characterize in the case of autonomous systems with evolving functionality (ASEFs). These are systems whose functionality—what they are meant to do, in fact do, and could do—evolve over time, typically in response to learning from environmental changes and at increasingly rapid timescales. For instance, an autonomous drone is an ASEF which continuously and dynamically reacts to obstacles in the environment en route to its destination, with little or no immediate input from human controllers beyond the initial instructions.

Although autonomous systems and AI are not exactly synonymous, their shared quality of being adaptive in response to the environment, particularly as a kind of ‘intelligence’, is of particular interest to investigating trust in technologies. The challenge of understanding trust and trustworthiness in the context of evolving functionality is that such systems are not predictable in the way that non-evolving systems are. The fact that they may change their goals, processes and outputs means that naïve or simple inductive trust is less possible and, rather, forms of trust that are more akin to human–human trust may be needed.^1^

While it is not intrinsically problematic for trust to be conceptually nebulous, as is the prerogative of language, the inclusion of trustworthiness as an explicit objective in public and corporate policies necessitates clarity of expectation and purpose. The lack of this is partly what leads Freiman to call trustworthy AI ‘conceptual nonsense’ (2022). While it is impractical to proscribe the use of the word trust, particularly as everyday terminology, we align ourselves with Freiman, who notes that epistemic confusion is not merely a problem for philosophers; with the potential for ethics washing and misalignment of expectations between different stakeholders serving as two potential problems in practice (Freiman 2022). The problems of moral agency and responsibility of developers pose particularly salient questions, and the appropriate bearing of responsibility can potentially be deranged by an inappropriate use of the trust concept.

As such, there is a need to seek conceptual clarity about trust and trustworthiness in the context of ASEFs. We are undertaking this work as part of the UKRI Trustworthy Autonomous Systems (TAS) Node on Evolving Functionality,^2^ working alongside robotics engineers. As such, our focus has been on these technologies as exemplar use cases.

1.3. Research questions.

The Adaptable Robots, Ethics, and Trust (ARET) study sought to engage with this need through empirical analysis of individuals’ real-world reasoning about trust in ASEFs and using this alongside philosophical reasoning to inform our conceptual understanding of trust in ASEFs.

Rather than simply stipulate the conceptions we think are correct, our approach has been to take a concurrent top-down and bottom-up approach, so that we can consider how these terms should be conceptualized and used, informed by an understanding of this language as an experienced phenomenon. In so doing, we are committing to the idea that the meaning of language (at least in part) can be found in its use; and that claims about how we ought to live can be productively informed by (if not derived directly from) knowledge about how we do live (Ives et al. 2017).

ARET asked the following research questions:

1. How do developers theorize and operationalize trust and trustworthiness during the development and deployment of ASEFs?
2. How and why do other stakeholders (including end users) place trust in autonomous systems and what are their requirements for trustworthiness?
3. How should an ASEF be developed and deployed to be not only trusted, but also trustworthy?

In this paper, we focus on answering questions 1 and 2, though we indicate how the answers to these questions might point toward an answer to question 3.

## Methods

A qualitative interview study was best suited to answering questions 1 and 2, as it gave us the opportunity to engage directly with participants and explore in depth their understandings of trust and trustworthiness in the context of their relationship with ASEFs.

### Recruitment and sampling

Interview data were gathered from three types of participants: ‘developers’, ‘end users’, and ‘other stakeholders’ from August 2021 to November 2022. Developers included academic and commercial developers, recruited through invitation emails and word of mouth within the TAS network. Purposive sampling was used to recruit developers with relevant knowledge about the development of swarm robots, soft robots, and UAVs. Snowball sampling was then used to recruit colleagues of these initial contacts to discover potential participants who have relevant knowledge of autonomous systems.

End users and other stakeholders included any individual who could be affected by autonomous systems, industrial end users who might procure and use autonomous systems in their business, and any other individual or industry who might have experience and insight that might be informative. They were recruited through social media platforms and through contacts in the TAS network.

### Interviews

A combination of online and in-person interviews were conducted. Interviews were semi-structured, employing topic guides to ensure coverage of relevant questions, while allowing flexibility to accommodate the direction participants wished to take. Interviews were transcribed verbatim.

To guide participants’ reasoning, three use cases of ASEFs were chosen: swarm robots (used for tasks such as storing and retrieving objects); soft robots (used for gripping and manipulating objects of varying shapes and tolerances); and UAVs (used for parcel delivery). These systems were selected as representative of the spectrum of technical challenges of system predictability, environmental uncertainty, and cost of failure that are encountered in all autonomous systems, while also encompassing a wide range of operational situations which require fundamentally different design approaches.

End users and other stakeholders were also given the choice of watching one of the three videos depicting a sample scenario of an ASEF: either swarm robots, soft robots, or UAVs. The videos provided brief toy model demonstrations of the technology, such as a swarm cloakroom, a soft robotic gripper for use in retail warehouses, and UAVs for parcel delivery, as well as an explanation of their evolving functionality being central to their ability to operate in dynamic environments. These scenarios both ensured participants were familiar with at least one technology and provided material for discussion. Participants were not asked to view more than one video, as we felt this would have been off-putting and created a barrier to participation.

#### Topic guides

**Table:** 

Developers	End users	Other stakeholders
Their understanding of ‘trust’ and ‘trustworthiness’ in their specific research area	Their understanding of ‘trust’ and ‘trustworthiness’ in relation to UAVs, swarm robots, and soft robots	The reasons for their interest in being partners of a project aiming to develop techniques and methods to specify, design, verify, validate, curate, and regulate emerging and future generations of trustworthy autonomous systems with evolving functionality
Their ethical concerns regarding trust and trustworthiness in the development and deployment of ASEFs	Their ethical concerns regarding trust and trustworthiness in using UAVs, swarm robots, and soft robots	Their understanding of ‘trust’ and ‘trustworthiness’ in relation to UAVs, swarm robots, and soft robots
Their understanding of ‘evolving functionality’ in their specific research area; the impact that evolving functionality has on their understanding of trust and trustworthiness and on their ethical concerns regarding trust and trustworthiness^3^	Their understanding of ‘evolving functionality’ of UAVs, swarm robots, and soft robots; the impact that evolving functionality has on their understanding of trust and trustworthiness and on their ethical concerns regarding trust and trustworthiness	Their ethical concerns regarding trust and trustworthiness in using UAVs, swarm robots, and soft robots
Their approaches and methods to operationalize trust and trustworthiness in the development and deployment stages		Their understanding of ‘evolving functionality’ of UAVs, swarm robots, and soft robots; the impact that evolving functionality has on their understanding of trust and trustworthiness and on their ethical concerns regarding trust and trustworthiness
The way they think of other trustors of their technologies, and of their responsibilities toward them		

### Analysis

An inductive approach was taken to data analysis, wherein iterative thematic analysis was conducted through multiple coding passes. In the first pass, initial codes were generated using open, descriptive, and pattern coding to capture meaningful concepts in the data. In the second pass, a mix of in vivo and ex vivo coding was used to generate themes. Focused, axial, and theoretical coding was used to identify primary themes, and locate relationships and hierarchies between themes, ultimately producing a list of core themes and leading to the development of explanatory theory.

## Findings

80% of participants were male and 85% identified as white, potentially reflecting the gender and racial demographics of the developer profession. A diverse age range was achieved across the sample.

**Table:** 

Category	Age	Ethnicity	Gender	Disability^4^
End user	18–29	White British	Cisgender man	No
End user	30–39	White British	Male	No
Developer	40–49	Mixed	Male	No
Developer	30–39	Mixed race	Male	No
Developer	18–29	White British	Male	No
Developer	30–39	White British	Male	No
Developer	40–49	White British	Male	No
Developer	18–29	White British	Male	No
End user	40–49	White British	Male	No
End user	40–49	White British	Female	No
End user	60–69	White British	Female	Yes
End user	50–59	White British	Male	No
End user	50–59	White British	Female	No
End user	40–49	White	Male	No
End user	30–39	White British	Male	No
End user	60–69	White British	Male	No
End user	30–39	White British	Male	Prefer not to say
Developer	30–39	White	Male	No
Developer	18–29	White British	Male	No
Other stakeholder	Prefer not to say	Prefer not to say	Prefer not to say	Prefer not to say

Participants reasoned about trust in ASEFs in ways that were generally consistent with different philosophical accounts of trust. Five accounts of trust were implicated, including *rational, affective*, *credentialist*, *norms-based*, and *relational* accounts.

### Rational trust

The *rational account* frames trust as a reasoned, often deductive, belief placed by the trustor in the trustee’s predicted future actions (Ryan 2020). This account views the trustor acting from a place of relative epistemic certainty in order to make reasoned and evidence-based predictions. A subset of this account is *experiential* trust, which involves trustors placing trust on the basis of their own observations and experiences of the trustee’s behavior as a proxy for future actions.

“[The end user] would probably only be able to fully trust something after they have collected their own experience.” (D3)

“Often, we trust something because we have seen it performing reliably […] My car has always worked up to today, so I’ll trust it tomorrow as well.” (EU4)

“[On using a fictional drone parcel delivery ‘Dronie’] I’d be like a hawk on the first couple of times it happened and then after that I’d be like, oh yeah, I’m using Dronie again […] we do learn to accept that sort of technological exchange reasonably quickly.” (EU3)

“[On the trustworthiness of robots] have there been trials, have there been evidence, have you got those reviews on the company to say this robot is excellent. I guess it would be down to me before I used the robot in that way to feel confident either through data or demonstrations or other consumer feedback around what that robot can do.” (EU5)

Rational trust, as expressed by participants, is primarily a form of reliance related to the user’s assessment of the technology’s functioning, performance, and its likely continuation. This assessment may be based on personal experience of the technology and/or testimony, and is generally rooted in evidence, based on experience of performance (either of the technology or the testifier), and predictions about the future based on that experience.

Some participants also stressed, to greater or lesser degrees, an understanding of the technology itself. One participant cited different types of technologies, of which they have differing levels of comprehension and corresponding levels of trust.

“[…] do you trust a car? […] they've been around for 100 odd years, and you've got a basic idea of how it works, because there's some kind of engine and then there's some seats and a steering wheel […]

[On swarm robots] Once you start getting into things that you go, ‘Well, I don't get how it works,’ because you've got a dozen things that are scurrying around, and they're clearly not crashing into each other, but they're moving at high speed. That's where you then start coming unstuck, because not only do you not know how to build it, but you can't even process how it's doing what it's doing […] you can see an input, you can see an output, but you don't understand the system in between […] the key thing is, if you don't understand it, maybe you don't trust it.” (EU7)

This type of trust is markedly different from the simple experience-based reliance characterized above. While also rational in nature, the basis of this form of trust is not past experience, but the relative explicability of the technology.

### Affective trust

Rational trust stands in contrast to *affective* trust, which is based on emotive states. The affective account involves a trustor who believes in the goodwill of the trustee toward them (Jones 1996; Ryan 2020). Trust forms on the basis of the perception of the trustee’s positive feelings toward the trustor.

Our findings suggest an additional affective component that could be incorporated into this account, which involves the positive feelings (or lack thereof) experienced by the trustor toward the trustee that influence the former’s trust in the latter. In this sense, affective trust is characterized by not only a belief in the trustee’s goodwill, but also a general warmth and positive appraisal of the trustee, leading to trust.

Both positive and negative affect are closely linked to the appearance, esthetic appeal and, particularly, the physical instantiation of ASEFs, with one developer noting explicitly how technology can be designed to elicit trust by manipulating these esthetics and exploiting affect:

“There are ways in which we can design robots so that they look trustworthy even if they're not. Because you can use a bit of deception and design the robots so that people may feel like the robot is more familiar […] virtual assistants things like Alexa for example: they have soothing voices, they sound very familiar, very reassuring and we may end up trusting these technologies, even though perhaps we have no idea how they're going to store our data, who they're going to share them with.” (D9)

Similarly, another participant noted their discomfort with a teddy-bear shaped robot (Robear, designed to work in care homes) because it exploits our tendency to feel positively toward endearing appearances and to place trust in anthropomorphic agents.

“I suppose a Japanese aesthetic has actually affected how we view robots, because they very much like to make things cute. Yes, I think that is a bit dangerous […]” (EU8)

If an ASEF can invoke positive feelings of safety and reassurance (for example, through mimicking trusted human characteristics), the trustor is more likely to trust. Conversely, where a technology invokes the opposite of these feelings, such as by appearing unnatural or unappealing, it is less likely to invoke affective trust—illustrated by one participant with reference to robot vacuum cleaners:

“I find it a bit weird seeing […] these vacuum cleaners that wander around the house […] I just find it a bit odd, I find it’s very alienating.” (EU3)

### Credentialist trust

In the *credentialist account* of trust, the trustor uses signals about the trustee’s credentials as a heuristic for the prediction of trustworthy behavior. Credentials are interpreted broadly but generally refer to reputation, whether personal or institutional; or qualifications, whether academic or professional. For example, one developer said that when a new product is launched, user trust is based not in the product but on the reputation of the seller.

“If it was the first time that they came up with this as a product or as a platform, then they would be depositing the trust not in the product, but more in whoever is selling it or whoever is recommending it […]” (D3)

This participant points out the reputational heuristic that people employ to assess the trustworthiness of technology, either individual or institutional (and it is worth noting the link here to rational trust, as this trust heuristic will be linked to experience of past performance of the trustee). The seeming simplicity of this point is enriched by another participant’s consideration of the interaction between trust heuristics and the trustworthiness of technology, when asked to reflect on the trustworthiness of drones.

“I’m just thinking about the difference between […] an NHS drone flying overhead and a military drone flying overhead […] which one would I trust more?” (EU3)

When prompted by the interviewer to consider whether different types of drone operators, such as the National Health Service (NHS) or the military, require different levels of trustworthiness, given the differences in what different drone providers may be carrying, the participant asserted:

“No, I think there should be trustworthiness across. It’s probably quite unlikely that the technology wouldn’t be used across all kinds of drones […] Whether I would trust a military drone is a different matter but it’s almost irrelevant, and also they should be using the very best technology as they should […] Let’s just say there were NHS drones flying around in the UK, you’d expect them to be the very best.” (EU3)

Reasoning about the trustworthiness of a drone by this participant appears to be multifaceted: both considering the reliability of the technology itself in delivering cargo safely, the intentions and objectives of the operator, such as military or medical; and the credentials of the operator, which determines how readily the participant might place trust in both the technology and the operator itself. This articulation of trust involves both a reliance on technology and a belief in the operator’s trustworthiness, largely founded in goodwill, beneficence, and moral intentions.

The use of credentials can be more straightforward, as in the case of D3’s reliance on corporate reputation, or more complex, as in the case of EU3, but in both cases, any reliance on credentials includes necessarily an appeal to past performance, which links to the rational account of trust. This begins to illustrate the complexity of trust as a thick concept (Williams 1985), which is loosely defined and requires contextual reasoning.

### Norms-based trust

The importance of context for trust is further borne out in the *norms-based account*, sometimes called the *normative* account (Coeckelbergh 2012). Participants often spoke of the significance of norms (social, moral, professional, and so on) leading to an expectation of trustworthy behavior; with the placing of trust being a default state inherent to particular societal contexts of market exchange and social reciprocity.

Participants were given the example of swarm robots for jacket storage in cloakrooms. When asked to reflect on why trust might be a relevant concern for novel robotic technologies, multiple participants referred to the default state of trust and the norms therein.

“Well, it relates to my trust of the theatre to provide good customer service, not my trust of the robots themselves.” (EU10)

“I suppose it’s because of the rules [the robots] are governed by, because we know if it’s an employee […] a person, we know they’re governed by the rules of the organisation but swarm robots don’t seem to have that […]” (EU11)

“If I give you, the cloakroom assistant, my jacket […] I assumptively trust that you're not going to take my nice new jacket, and accidentally lose it […] or give it to your friend […] I also maybe assume that if it does go missing that the wrong jacket gets picked up [by the robot], then there's someone else there to step in and correct it.” (EU4)

These quotes reveal participants’ reliance on the role of social norms, those disincentivizing theft in this case, to induce the trustworthy behavior of others. Even where novel technological agents are implicated, which may be able to accord with but certainly cannot understand, social norms, participants nevertheless rely on the norms governing the behavior of the technology’s creators and deployers, ultimately placing their trust in the human owners and operators of systems.

In this context of the cloakroom providing a specific service, participants spoke of trust not in affective or credentialist terms, but in terms of behavioral norms. They understood the relationship between a buyer and seller in a market to be grounded in shared understandings and subject to boundaries of acceptable and unacceptable behaviors. Although these norms are often implicit and fuzzy, they are nevertheless assumed and relied upon to trust. EU10 encapsulates the relationship of norms-based trust well in the following:

“I think that one of the things that’s most interesting is what you said to me: why is trustworthiness of such concern in these autonomous systems, whereas it’s not in human systems? I think that’s because, in human systems, we have norms of what to do if things go wrong. Now that does vary culturally, over history and from country to country […] None of that exists for robots and we don’t even know how to create it, because the robots aren’t intelligent enough, so we’ve got no idea of how to establish trust with them.” (EU10)

In their final sentence, EU10 highlights the crux of the matter: how trust can be established between humans and autonomous systems, and suggests that it is perhaps the robot’s insufficient intelligence which precludes a relationship of trust. The implication is that a robot with specific local abilities and low generalized intelligence does not seem an appropriate target for a relationship of trust in the way that a human agent, capable of moral reasoning and comprehending shared social norms, certainly is.

### Relational trust

When considering the trustworthiness of technology, participants often referred to the need for accountability. Discussion of technological safety, particularly failure modes, led to many participants talking about moral responsibility and the need for identifiable, accountable moral agents into which trust could be deposited, constituting a *relational* trust. This relationship was sometimes said to supersede reliance on the technology itself, and to be central for trustworthiness.

#### Agency and reliance

One participant claimed that they might trust a robotic cloakroom system more than a human attendant, based on the fact that robots lack agency or the potential for deliberate malice.

“[On trusting cloakroom robots] Because I know that the robots aren’t going to steal stuff out my pockets … which I do worry about when I put my coat in a cloakroom at venues […] I think to a certain extent it is my knowledge of how robots work […] unless the system designer specifically puts something malicious in there, the way machines work is they only do, even the small robots, they can only do what they’ve been told to do in the first place and nothing more.” (D6)

This quote demonstrates a *reliance* on the proper functioning of the cloakroom robot, which is incapable of transcending the boundaries of its programming and can be relied upon to carry out tasks with the potential for relatively quotidian errors. The robots’ lack of intrinsic motivation precludes the possibility of catastrophic deviations, such as the desire to steal.^5^

In contrast, another participant reflects on how it is precisely this lack of intrinsic motivation which leads to their distrust.

“[…] I’m a human and I know what it’s like to be a human. When human beings do things, it’s usually for a reason. Sometimes we don’t always know what the reason is […] but they nearly always do have a reason that makes sense to them, and I don’t know what motivations a machine learning drone has. I don’t know why it’s doing anything so there’s just that sort of uneasiness like if you don’t know what something wants or whether it even does want anything or is capable of desire […] If you understand what somebody wants and you want them to act fairly consistently in pursuit of that goal and that makes them relatively not predictable but reliable […] people kind of make sense usually you know, sociopaths aside, whereas robots I don’t know.” (EU1)

Although D6 and EU1 may diverge in how they perceive a robot’s lack of agency, they both describe the importance of understanding an agent’s behavior as a prelude to trust. D6 feels that they understand the constraints of robotic behavior, while EU1 reflects that they might not intuitively grasp the behavior of a robot in the same way they can a human’s. It is clear that behavioral explicability is central both to the rational account of trust and to facilitate one’s understanding of and subsequent *reliance* on technology.

#### Trust beyond reliance

Other participants spoke of trust beyond reliance on functioning. A few participants reflected on how the lack of an identifiable and accountable moral agent, responsible for the behavior and particularly failure of technology, engenders suspicion and distrust, which is to be distinguished from any assessment of the technology’s reliability.

“[…] with the drone element […] I’m not sure whether it’s the technology that I don’t trust or the person controlling the technology […] So for about a week last summer somebody kept flying a drone over the fields around the back of our house and coming very close to our house and there’s an element of suspicion there about, what is that drone, what it is doing, what is it capable of doing […] you can't see the operator and it doesn’t feel right.” (EU3)

“I suppose my concern […] is that I'm handing over a lot of data for which I've got no idea who that data is going to. […] it's the facelessness of where that data is going, and the lack of accountability for it.” (EU8)

#### Moral responsibility

Without engaging in debate about moral agency for non-human or artificial agents, it was generally acknowledged by participants that moral responsibility and agency is only held by human beings, and not by even the most highly complex and autonomous systems. In fact, in the case of highly complex systems, the need for an accountable agent seemed to be even more significant, given the lack of user understanding of the system and how it might behave.

“[…] in everyday life we do use the word ‘trust’ for things like… I might […] have to cross a river on a bridge. I might say, ‘The bridge looks very shaky.’ I may say, ‘Do I trust this bridge to take our weight? Will I walk over it? Will I take my car over it?’ […] again, we come back to the judgement of human beings, which I think is why the legal system always tries to bring things back to a liable human who has responsibility […]” (EU10)

“[On autonomous vehicles] At some point […] there are enough things in the car that are now doing the workload and I don’t know where that point is by the way […] but doing enough of the workload that if something goes wrong and my Tesla […] is in autonomous mode and I had my hands on the wheel […] but it still had a crash, whose fault? And so somewhere in this decision-making process about fault and accountability or in this spectrum there is a tipping point […]” (OS1)

“[On user understanding of systems] It’s kind of pie in the sky these days with that because it’s very unlikely that end users are going to be able to understand the whole thing but they might understand principles behind it. […] You have to devolve your trust don’t you, to the developer in this.” (D12)

OS1 acknowledges the challenge in allocating moral responsibility in the case of a highly autonomous system such as a fully autonomous vehicle, in which a threshold might exist (in principle) for the absolution of human responsibility, given a system with sufficient agency. A few other participants approach this problem differently by highlighting the inevitability of human responsibility.

“Any robotic system will have a human or legal entity as an owner, ultimately, and as an operator […] we’re trying to remove humans from the system – that’s the problem. If we re-insert a human, we re-insert a person to trust and some liability.” (EU10)

“[On drone failure modes] with a drone where there’s no pilot […] but I don’t think you can go back to the person who wrote the code […] I don’t think that’s reasonable […] although the code should have been better […] I’m sure there would be all sorts of litigations going on to make sure the company never did it again, but ultimately it’s the company as a whole, there’s like a collective responsibility there. If you were looking to profit off this sort of technology as well, there has to be like a collective responsibility for if it goes wrong that your company is going to have to pay.” (EU3)

“There’s an interesting analogy, though, with working animals, so to what extent do we trust an animal? […] ultimately, if a dog or animal misbehaves, the human owner is liable.” (EU10)

“[…] rather than thinking that robotic systems have to be inherently trustworthy, we should start to think of them as like human beings, who are not necessarily very trustworthy, and so we have all sorts of rules about criminality, liability […] When a human does something slightly wrong, we all just get grumpy and they’re not our friend anymore, or there’s more minor social sanctions. What are the sanctions for robots?” (EU10)

In comparing robots to animals and reflecting on the difficulty of sanctioning a robot, EU10 seems to be hinting at the necessity of human responsibility given the impossibility of allocating moral responsibility to non-humans. Robots, as non-humans, do not appear to possess the capacity for moral agency. While moral agency and its theoretical determinants is by no means a resolved question, participants naturally referred to moral responsibility and legal mechanisms for accountability in the same breath.

#### Accountability

In discussing accountability, participants spoke most about developer accountability, as enforced through liability and regulatory mechanisms. One challenge—the allocation of responsibility across agents in the supply chain—received particular attention. A few participants spoke of concerns about their own responsibility for the use of technologies which they developed, the deployment of which they had no control over. This development-deployment gap was of particular personal concern to developers.

“[On developing drones] There’s a few examples that I think are really clear that I’m always concerned about, which is the potential of these systems being used as killing machines, for instance, so that’s something that I’m always aware of, I’m always struggling with, at least internally. I don’t know even how to defend that, because I still do it […] do you have to stop? Or you just have to do your work and then just don’t engage with those? Or you have to engage with those and make sure that they are developed in a responsible way?” (D1)

“[On the development-deployment gap] Without knowing what [users are] using it for, is it wrong of me to have provided them with something that they can then unethically use, or should they have a responsibility to use it ethically if I can give them accurate […] results that they can trust […] it really depends on where in the pipeline I’m selling it. If I’m an engineer and I’ve made a tool and that’s as far as I can go, then I probably don’t think it’s fair to expect this kind of bracketing, this like well, 20% [safety] is as low as you should possibly go in any scenario, but if you’re then the manufacturer, and you’re giving this to people for specific, bespoke use cases, maybe in that scenario you should be drawing a ring around the areas that they should be using and saying […] I’m not going to let you go outside of this ring because that would be bad. I think it depends really on where in the chain you are […] ” (D10)

A few participants asserted that the moral responsibility of deployment ought to fall squarely on the deployers, provided that the developers had done their own due diligence on technological performance.

“When I was working a lot with software guys back in the early 90s, they drove me mad. They were doing all this stupid stuff, that wasn't any use for the end user. But then I realised it's not their job to do that, they're seeing what's possible, and other people need to decide what's sensible, reasonable, moral and all the rest of it. It's not their job to do that.” (EU8)

“Once we have developed a system, be very specific, very clear. Even with all of the effort and work that I put into this, I am aware that this system only works under these parameters, on the conditions that I’ve tested. This is the work of somebody else, or my work in the future, to try and develop ways in which I could test this further to make sure it is trustworthy for all the conditions.” (D3)

“We have verified this module of software or hardware […] That is just that bit that you develop. You don’t know what kind of complexity is going to arise from the whole.” (D5)

“[…] you’re also taking some responsibility because you’re saying I’ve tested these, I’ve made sure that these fit within bounds of safety, and I’m not saying let them free and just purely trust in the system, you’re probably still going to be there with a big emergency stop button, but if you exist within this parameter scape, you should be able to meet some minimum safety requirements that have been defined. You should be able to meet some minimum ethical requirements that have been defined and if you go outside of these bounds, I can’t tell you what’s going to happen, I’ve not tested that.” (D10)

Similarly, in considering the use of cloakroom robots, a few of the participants used compensation as an example of how they would place their trust in the human agent responsible for an autonomous system, highlighting both the significance of developer accountability and of identifiable moral agents.

“[…] taking the example of the cloakroom, if as I arrived at the cloakroom they said, ‘We’ve got this robotic cloakroom system, but if anything goes wrong, we promise to buy you a brand-new coat within ten days,’ I’d say, ‘Fine.’ I’ve got nothing to lose—no problem, right? So, what I’ve done there, I’m not trusting the robots; I’m trusting the human operator of the robots, because they’ve made an explicit promise.” (EU10)

“[On drones causing injuries to pedestrians] I think the operator would need to be held responsible […] the investigation would need to be done by the CAA […] depending on the person’s injury, they may need medical attention and compensation […]” (EU12)

It was often acknowledged that systemic regulation was needed, utilizing incentives and sanctions to motivate responsible innovation. On the example of a drone collision causing injury to a pedestrian, several participants spoke of third-party accountability mechanisms and fiduciary-type relationships to facilitate trust, such as assurance and redress. The use of a trusted agent as a third party that would, for example, secure compensation, conduct investigations, or regulate corporate behavior, serves as a critical component of a larger context in which participants would be willing to place their trust.

“[…] so two different companies, the drones collide, so they’re probably going to argue over whose drone was at fault, and it’s hit you, so you don’t really care whose drone is at fault, you just know that you got hit. […] I guess the question in the result of injury and/or death is who is going to pay? […] surely there would have to be some sort of public liability insurance or even insurance against damage to building and property, for anything that’s whizzing around in the sky.” (EU3)

“[…] what I would want to do is go back to my company and say, ‘This went wrong. Can you pursue this case for me?’ And I would have thought my company would then go to the soft robot company and say, ‘Pay damages,’ or whatever it may be […] If softrobot.co.uk or whatever it is said, ‘Oh, it’s nothing to do with us because we just bought in the AI from another company’ […] I don’t care one jot about that because my trusted organisation will pursue it for me, so it doesn’t matter how the law deals with it or how the companies deal with it […] because I have someone on my side. I have an organisation that is backing me, that is pursuing my interests, and will do that for me. I can sit back—maybe I’ve broken my hip—and let my organisation get the compensation for me or get my hip replaced, or whatever it may be.” (EU9)

The presence of regulatory mechanisms is cited as not only providing material protection to consumers but is recognized by end users to present a credible signal that their interests are being protected. One participant, OS1, addresses this protection, or (perceived) *value alignment*, more explicitly, from an industry point of view.

“[…] at its heart, insurance is a promise to put you back to where you were after an incident […] You buy the product hoping never to use it, if you do use it you, you want to be able to trust it’s going to work: as in the promise will be fulfilled. And then the insurers’ job is not to try and get out of it, but to make sure that that is valid and that it is fair because otherwise you’re penalising the other people in the insurance pool. So, at its heart, the insurance is about designing trust and trustworthiness in what we do.”

Users and developers also addressed value alignment from a user point of view.

“[AI] affects lives, so [AI applications] should have people’s best interests in mind when they’re made.” (EU1)

“[On values of developers] there is an important challenge there because when we build software... okay it should be ethical... but what [values] is behind these? We also need to think of what values should influence our models and all the decision making in those systems.” (D4)

By perceived value alignment, we are not referring to the alignment of AI systems with human interests, as is a terminological convention in the technical literature concerning advanced AI (Gabriel 2020). We refer, rather, to the user’s perception that their interests are being protected by a broader system at work, whether sociotechnical, institutional, legal, or political, which allows them to feel that their values and interests are respected and protected.

## Limitations

Our study is limited by the ethnic and gender homogeneity of the sample, which likely reflects the self-selected nature of the participants (recruited through snowball sampling and networks), as well as the demographic characteristics of the robotics profession in the UK.^6^

As a result, the generalizability of some of our findings may be somewhat limited. To address this issue, we issue two caveats; first, we do not suggest that the views expressed by our participants on trustworthiness are universal or representative of the general population. Second, we also intend for these findings to inform thinking on trustworthy AI and to connect with existing debate in the philosophical literature, as opposed to perfectly representing the views of the public.

## Discussion

The ARET study sought to contribute to philosophical accounts of trust in AI and ASEFs through an empirical exploration of how different stakeholders conceptualize, reason about, and place trust in these systems.

The question of a gap between trust and trustworthiness was of interest, as the use of ‘trust’ and ‘trustworthy’ do not always distinguish between (1) the property of being deserving of trust as a piece of functioning technology; (2) the quality of being deserving of trust as a morally responsible human operator; or (3) of inducing user trust by allaying public fears, demonstrating corporate goodwill, or otherwise. These differing operationalizations of trust and trustworthiness can be directly contradictory if, for example, developers exploited trust heuristics to induce public trust in their technologies.

As such, the ambiguity of trust as a concept in sociotechnical systems, with confusions over how trustworthiness of a technology is achieved and how ‘trust’ operates as a polysemous device, was the motivation for conducting this study, which draws on participants’ real-world reasoning in the context of specific use cases of ASEFs.

Before discussing our findings specifically, it is important to situate them in the context of ongoing disagreements surrounding the language of ‘trustworthy AI’ in the philosophical literature. Following trust theorists in the 1980s and 90s, who wrote at length about the philosophical underpinnings of trust as a general concept (see, among others Baier 1986; Hawley 2014; Luhmann 1988), a wave of theorists of trust in technology in the 2010s and 20s rejected the notion of trustworthy AI and, at times, advocated for *reliance* on AI instead of trust, which is said to exclusively inhabit the domain of human relationships (Braun et al. 2021; Bryson, cited in Lewis and Marsh 2022; Nickel et al. 2010; Ryan 2020; Sutrop 2019; Taddeo et al. 2019; Tallant 2019).

The central thrust of many of these arguments is that the granting of trust must involve a degree of belief in the future actions of the trustee, which are positively aligned with the trustor’s interests and which involves both a vulnerability that this trust might be broken and an expectation based not merely on the trustee’s previous behavior, but also social conventions, norms, commitments and moral responsibility. It is for this latter reason in particular that some trust theorists have asserted that one cannot truly trust AI, however autonomous and seemingly agential.

In more recent years, some theorists have attempted to make more moderate claims about trustworthy AI, by considering how the flexibility of language and the agent-like quality of increasingly advanced AI allow AI to serve as a candidate for trust. Simion and Kelp (2023) argue that conceptualizing trust as incurring and fulfilling appropriate commitments can be applied to AI, which is considered trustworthy if it fulfills its design functions, as specified by a developer, and its etiological functions, which are expectations of its functioning based on historical norms of the use context. Ultimately, AI is said to acquire a set of norms on this basis which govern its functioning and the expectations of trustors.

In response to Simion and Kelp, Nyrup constructs a ‘modest anthropocentric’ account of trust, refusing to grant that AI is automatically trustworthy if it fulfills design and etiological functions, as these may not be sufficient to align with a user’s fundamental interests (2023). He agrees that trustworthy AI is possible on the basis of the fulfillment of commitments, but also claims that these commitments ought to be derived from the role that the AI system plays in social practices into which it is embedded, such as the medical context in the case of a diagnostic AI.

Lewis and Marsh also agree that trustworthy AI is plausible but take a slightly different approach from the commitment framing of trust (2022). They employ a functionalist account, seeking to define trustworthy AI on the basis of its purposes and alignment with user goals. AI is thought to be trustworthy if it achieves some combination of the following features: competence, predictability, integrity, and willingness. These features are additive rather than singularly essential for an assessment of trustworthiness.

Lewis and Marsh in particular stipulate that this account stems from a pragmatic view of trust, which understands that individuals will continue to employ the language and heuristics of trust, regardless of how philosophers construct idealized accounts (2022). The examples of animals, young children, and other machines are used to illustrate how individuals instinctively use the language of trust for trustees which are not necessarily full moral agents into which to deposit trust.

Although all of these accounts, both anthropocentric objections and moderate trustworthy AI claims, can be differentiated in minor and precise ways, they generally come to a central divergence on whether it is appropriate or even possible for AI to occupy the position of ‘trustee’, given that it can, *to an extent*, embody human values, act autonomously, and act with purpose in service of those values in a way that trustors would recognize as agential in nature. ASEFs, in particular, might be particularly contentious candidates, given their ability to adapt in dynamic environments.

Without seeking to answer these questions definitively or in the abstract, the ARET study aimed to make a contribution to this debate by exploring how individuals intuitively reason about trust. As trust is a widely used concept, the views of citizens and philosophers alike ought to be considered when formalizing, or rejecting, ‘trustworthy AI’.

It was found that accounts of trust drawn from existing epistemological literature broadly align with how both end users and developers of ASEFs place trust and specify requirements for trustworthiness. Participants spoke of their reliance on various heuristics to measure trustworthiness of ASEFs, and their broader sociotechnical context, primarily including empirical evidence, personal experience, emotional affect, and societal and contextual norms. These heuristics correspond with various accounts of trust, including the rational, affective, credentialist, and norms-based accounts respectively.

It was also revealed that these heuristics are at times in conflict with one another, with differing implications for the allocation and perception of moral responsibility and agency in the case of technological failure. For instance, when faced with a variety of signals about trustworthiness, some relating to the performance of the ASEF itself and some relating to the behavior of human operators or owners, it is not clear which heuristics ought to be prioritized. This somewhat stochastic landscape of trust heuristics is belied by the consistency with which participants referred to human accountability, which leads us to a relational account of trust.

Two key elements of the relational account of trust are as follows: identifiable moral agents and value alignment. In simple terms, individuals need to have someone to hold responsible and someone who has their (ideally best) interests at heart, in order to perceive this someone (or something associated with that someone) as trustworthy. Although participants understood the challenge of allocating precise responsibility, particularly in the case of liability, complex supply chains, and highly autonomous systems, they nevertheless converged on the importance and indeed inevitability of accountable human agents, whom are then quite naturally linked with existing institutions and duties of legal responsibility and redress.

The relational account of trust also has implications for the meaning of reliance on technology. Participants, particularly developers, often spoke of the desirable technical elements which contribute to one’s reliance on the continued performance of an ASEF, such as explainability, safety and robustness, concepts intimately familiar to AI and sociotechnical scholars*.* However, they were also acutely aware of the delimitations of reliance and how trust is a broader and at times altogether divergent concept; one which speaks to the qualities of human operators and the ‘socio’ of sociotechnical systems. While the term ‘trust’ is used interchangeably to refer to reliance on technical systems and to trust in human operators, the conceptual distinction was nevertheless made by many participants, with one asserting that trust is in fact impossible between a person and an artefact (EU9).

**Fig. 1:**
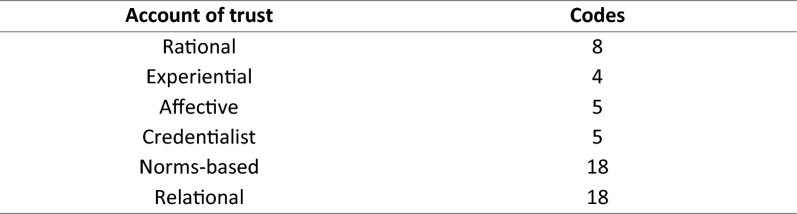
The number of codes assigned to each heuristic of trust

Figure 1 demonstrates the particular significance of relationships between the trustor and an identifiable moral agent as the trustee. The rational and experiential accounts of trust receive a moderate number of combined citations, highlighting the significance of reliance on and empirical understanding of ASEFs. However, it is the norms-based and relational accounts which receive the highest number of citations and are both inherently linked to human agency, society, and convention. Ultimately, this distribution of codes demonstrates the particular significance of human relationships for trust as a primarily, perhaps exclusively, social endeavor.

Once an appropriate moral agent has been identified for a trustor to deposit their trust in, the trustor then needs some assurance that the trustee will act in their interest. A credible signal of acting in the trustee’s interest is not straightforward to define, and we do not have data that allows us to comment meaningfully on this. However, what is clearly borne out in this corpus of interview data is that the question of value alignment is central in the eyes of end users when they infer the trustworthiness of agents.

Various mechanisms to secure and demonstrate value alignment were cited by participants, including legal and institutional mechanisms such as third-party assurance, insurance systems, redress and compensation, and the use of sanctions against corporate malpractice. Generally speaking, the precise details of these mechanisms were not significant to participants in the abstract but formed a central part of their assessment of trustworthiness by presenting a clear signal of value alignment, with the additional potential for punitive action against agents who betray consumer trust.

Value alignment and moral agents are intertwined as artifacts cannot accept moral responsibility and sanction, or indeed comprehend shared values. They can only be deployed by those agents that are capable of accepting and wielding such things (Bryson, cited in Lewis and Marsh 2022). A sophisticated technology could be said to embody certain values, but it does not embody them to the extent that it can be held responsible for them. One significant problem, then, may be that until and unless artificial agents can be considered moral agents, it seems implausible that they could be held responsible for their actions, and, on the accounts of trust demonstrated in our data, might not be appropriate recipients of trust.

## Conclusion

Despite ongoing debates on machine agency or even questions about their potential consciousness (Dennett 2006; Torrance 2008; O’Grady et al. 2022), it is not appropriate, given current technological capacities, to ascribe moral responsibility to AI systems. As technological progress marches on, the prospect of increasingly and fully autonomous systems will require yet more reflection on whether they are fit for purpose as moral agents and, in this sense, be fully deserving of trust language. Although disagreement over AI agency reigns, some consensus is likely found in the claim that we have not yet reached this point.

We acknowledge that some may consider it best to avoid the word trust and instead to speak of reliance in systems and to reserve trust for their operators, a point which has been convincingly argued by some (Ryan 2020; Kerasidou et al. 2022). The use of “appropriate confidence” terminology has also been suggested by the NHS AI Lab to illustrate a continuous degree of confidence (or lack thereof) in healthcare technologies: as liable to change, rather than a binary trust state (2022).

However, it is clear that individuals will continue to intuitively reason about trusting AI, regardless of their ultimate capacities, just as they do about other machines, animals, and young children, whose moral responsibilities and capacity to act autonomously on par with human adults may also be in question. Our interview study has provided further evidence that both lay users and developers use the language of trust rather reflexively in reference to machines and their functional reliability, but that they also use trust in a fundamentally relational manner which centers human beings as the ultimate holders and arbiters of moral responsibility.

Care ought to be taken when trust language is used to avoid equivocation on its meaning and purpose, such as to distinguish between its use to indicate the functioning of machines or the accountability of their creators. In light of the flexibility and imprecision of everyday language, it seems impractical to recommend an outright foreclosure of terminology. It would be impractical to proscribe the use of trust language in the context of AI.

Given the potential for ongoing disagreement on linguistic matters, it seems appropriate to end on a note of relative consensus. Trust is tightly linked to accountability in its various forms: moral, legal, and social. It is unsurprising that the clearest refrain echoed by participants in this study was the need for developers and other relevant human actors to be held accountable for the actions of AI systems, as is in line with the regulatory priorities of the UK government (Department for Science, Innovation and Technology, 2023). Regardless of where one stands on debates surrounding terminology, this central point found in our data seems to be the most incontrovertible, although questions of implementation will no doubt prove a challenge, particularly as AI systems become more adaptive and autonomous.

## Data Availability

As the participants of this study did not give written consent for their data to be shared publicly, supporting data is not available.

## Footnotes

1 However, we are not suggesting that ASEFs are human or have human properties, simply that increasingly autonomous systems may induce forms of trust and thinking about trust which departs from our conventional understanding of ‘trust’ and reliance in machines.

2 https://tasfunctionality.bristol.ac.uk/

3 While the question of evolving functionality was discussed with participants, it was generally not a compelling concept for participants with regards to the topic of trust. As such, evolving functionality is not a significant feature of our analysis, although we do discuss the autonomy and complexity of systems.

4 Our sampling strategy sought to be actively inclusive, and we asked for this data so that we could purposively sample and include participants who have a disability, especially if they felt it gave them particular insight into the research topic.

5 It is worth noting, however, that the robot characterized by D6 does not appear to have evolving functionality.

6 While specific statistics about gender and racial demographics in the robotics profession is not readily available, some sources point to lower proportions of women in STEM and the AI profession more broadly (see for example World Economic Forum 2018).

## References

[CR1] Baier A (1986) Trust and antitrust. Ethics 96(2):231–260. 10.1086/292745

[CR2] Braun M, Bleher H, Hummel P (2021) A leap of faith: is there a formula for “trustworthy” AI? Hastings Center Rep 51(3):17–22. 10.1002/hast.1207 10.1002/hast.1207 33606288

[CR3] Brundage, M. et al (2020) ‘Toward trustworthy AI development: mechanisms for supporting verifiable claims’. Available at: 10.48550/ARXIV.2004.07213.

[CR4] Coeckelbergh M (2012) Can we trust robots? Ethics Inf Technol 14(1):53–60. 10.1007/s10676-011-9279-1

[CR5] Dennett D (2006) Cognitive wheels: the frame problem of AI’. In: Philosophy of psychology: contemporary readings. Taylor and Francis Group, Routledge. London, UK

[CR6] Department for Science, Innovation and Technology (2023) A pro-innovation approach to AI regulation. London, UK. Available at https://www.gov.uk/government/publications/ai-regulation-a-pro-innovation-approach/white-paper

[CR7] Freiman O (2022) ‘Making sense of the conceptual nonsense “trustworthy AI.”’ AI Ethics. 10.1007/s43681-022-00241-w

[CR8] Gabriel I (2020) Artificial intelligence, values, and alignment. Minds Mach 30(3):411–437. 10.1007/s11023-020-09539-2

[CR9] Hawley K (2014) Trust, distrust and commitment: trust distrust and commitment. Noûs 48(1):1–20. 10.1111/nous.12000

[CR10] Independent High-Level Expert Group on Artificial Intelligence (2019) Ethics guidelines for trustworthy AI. European Commission

[CR11] Ives J, Dunn M, Cribb A (eds) (2017) Empirical Bioethics: Theoretical and Practical Perspectives, 1st edn. Cambridge University Press, Cambridge. doi: 10.1017/9781139939829.

[CR12] Jones K (1996) Trust as an affective attitude. Ethics 107(1):4–25. 10.1086/233694

[CR13] Kerasidou C et al (2022) Before and beyond trust: reliance in medical AI. J Med Ethics 48(11):852–856. 10.1136/medethics-2020-107095 34426519 10.1136/medethics-2020-107095 PMC9626908

[CR14] Lewis PR, Marsh S (2022) What is it like to trust a rock? A functionalist perspective on trust and trustworthiness in artificial intelligence. Cogn Syst Res 72:33–49. 10.1016/j.cogsys.2021.11.001

[CR15] Luhmann N (1988) ‘Familiarity, confidence, trust: problems and alternatives. In: Gambetta D (ed) Trust: making and breaking of cooperative relations. Basil Blackwell, Oxford (Preprint). Available at: http://citeseer.ist.psu.edu/luhmann00familiarity.html.

[CR16] Mökander J, Floridi L (2021) ‘Ethics-based auditing to develop trustworthy AI. Minds Mach 31(2):323–327. 10.1007/s11023-021-09557-8

[CR17] NHS AI Lab and Health Education England (2022) Understanding healthcare workers’ confidence in artificial intelligence (AI) (Part 1)

[CR18] Nickel PJ, Franssen M, Kroes P (2010) ‘Can we make sense of the notion of trustworthy technology? Knowl Technol Policy 23(3–4):429–444. 10.1007/s12130-010-9124-6

[CR19] O’Grady KL et al (2022) ‘Trust, Ethics, Consciousness, and Artificial Intelligence’. In: 2022 IEEE/AIAA 41st Digital Avionics Systems Conference (DASC). 2022 IEEE/AIAA 41st Digital Avionics Systems Conference (DASC), Portsmouth, VA, USA: IEEE, pp 1–9. doi: 10.1109/DASC55683.2022.9925874

[CR20] Reinhardt K (2023) Trust and trustworthiness in AI ethics. AI Ethics 3(3):735–744. 10.1007/s43681-022-00200-5

[CR21] Ryan M (2020) In AI we trust: ethics, artificial intelligence, and reliability. Sci Eng Ethics 26(5):2749–2767. 10.1007/s11948-020-00228-y 32524425 10.1007/s11948-020-00228-y PMC7550313

[CR22] Simion M, Kelp C (2023) Trustworthy artificial intelligence. Asian J Philos 2(1):8. 10.1007/s44204-023-00063-5

[CR23] Smith DH et al (2023a) ‘Ethics of trust/worthiness in autonomous systems: a scoping review.’ In: Proceedings of the First International Symposium on Trustworthy Autonomous Systems. TAS ’23: First International Symposium on Trustworthy Autonomous Systems, Edinburgh United Kingdom: ACM, pp 1–15. doi: 10.1145/3597512.3600207

[CR24] Smith DH et al (2023b) ‘Ethics of trust/worthiness in autonomous systems: a scoping review. In: Proceedings of the First International Symposium on Trustworthy Autonomous Systems. TAS ’23: First International Symposium on Trustworthy Autonomous Systems, Edinburgh United Kingdom: ACM, pp. 1–15. doi: 10.1145/3597512.3600207.

[CR25] Sutrop M (2019) Should we trust artificial intelligence? Trames J Human Soc Sci 23(4):499. 10.3176/tr.2019.4.07

[CR26] Taddeo M, McCutcheon T, Floridi L (2019) ‘Trusting artificial intelligence in cybersecurity is a double-edged sword. Nat Mach Intell 1(12):557–560. 10.1038/s42256-019-0109-1

[CR27] Tallant J (2019) You can trust the ladder, but you shouldn’t. Theoria 85(2):102–118. 10.1111/theo.12177

[CR28] Torrance S (2008) ‘Ethics and consciousness in artificial agents. AI Soc 22(4):495–521. 10.1007/s00146-007-0091-8

[CR29] World Economic Forum (2018) The global gender gap report 2018. Switzerland, Geneva

[CR30] Williams B (1985) Ethics and the limits of philosophy. Routledge, London

